# Pathophysiology of Femoral Fractures in Hypophosphatasia

**DOI:** 10.1007/s11914-025-00929-y

**Published:** 2025-09-04

**Authors:** Fabiana G.A. Tabegna, Mark Garton, Simona D’Amore, Linda Skingle, Scott Dillon, Melinda J. Duer, Gavin P.R. Clunie, Kenneth E. S. Poole

**Affiliations:** 1 NIHR Cambridge Biomedical Research Centre, Department of Medicine, Addenbrooke’s Hospital, University of Cambridge, Cambridge, United Kingdom; 2 Wrexham Glyndwr University, Clywd & Nuffield Health, Wrexham, Shrewsbury, Shropshire United Kingdom; 3 Department of Precision and Regenerative Medicine -Ionian Pole, School of Medicine, Aldo Moro University of Bari, Bari, Italy; 4 Yusuf Hamied Department of Chemistry, University of Cambridge, Cambridge, United Kingdom; 5 Department of Rheumatology, Addenbrooke’s Hospital, University of Cambridge, Cambridge, United Kingdom

**Keywords:** Hypophosphatasia, TNAP, Femoral fractures, Osteomalacia, Osteopetrosis, Bone mineralization

## Abstract

**Purpose of Review:**

In this review, we will examine the pathophysiology, anatomy, biochemistry, and genotype-phenotype correlation of femoral fractures in adult hypophosphatasia.

**Recent Findings:**

Hypophosphatasia (HPP) is a rare genetic disease characterized by low activity of tissue-nonspecific alkaline phosphatase (TNAP). The disease presents a broad spectrum of clinical manifestations primarily determined by the degree of residual TNAP activity. Adults with HPP of moderate clinical severity may present with spontaneous femoral fractures that are like the atypical femoral fractures (AFF) of long-term bisphosphonates users. In this review, we will focus on the paradox that while HPP can cause biopsy-proven osteomalacia (pathologically impaired bone mineralisation), the spontaneous femoral fractures that characterise adult HPP do not exhibit typical osteomalacia features. Instead, they resemble the femoral fractures that occur in other diseases such as osteopetrosis where bone becomes excessively dense, brittle and highly mineralised due to osteoclast dysfunction.

**Summary:**

This review examines the key aspects of the pathophysiology of femoral fractures in adults with HPP, offering new insights into the role of anatomical, molecular and biochemical bone abnormalities that characterise the disease. Further investigations of HPP patients with femoral fracture are needed to examine the nanoscale crystal structure of the bone and to study abnormalities in fracture healing and bone resorption.

## Introduction

Hypophosphatasia (HPP) is a rare inherited metabolic disease characterized by low activity of tissue-nonspecific alkaline phosphatase (TNAP) due to pathogenic variants in the *ALPL* gene [1]. TNAP is a widely distributed, functionally pleiotropic enzyme, deficiency of which can cause several debilitating effects, primarily impacting bone. HPP encompasses a wide spectrum of manifestations and severity, ranging from stillbirth (due to the catastrophic impact of severe osteomalacia in utero) to childhood and adulthood bone pain, premature loss of teeth and diverse musculoskeletal abnormalities that include fractures (Table 1) [2–6]. The spontaneous fracture of one or both femoral shafts in HPP is a particularly alarming presenting feature in adults [7]. HPP-related spontaneous fractures of the femoral shaft are an enigmatic skeletal complication usually seen in adults with HPP of moderate clinical severity and first reported in an adult case from 1976 [8].

**Fig. 1:**
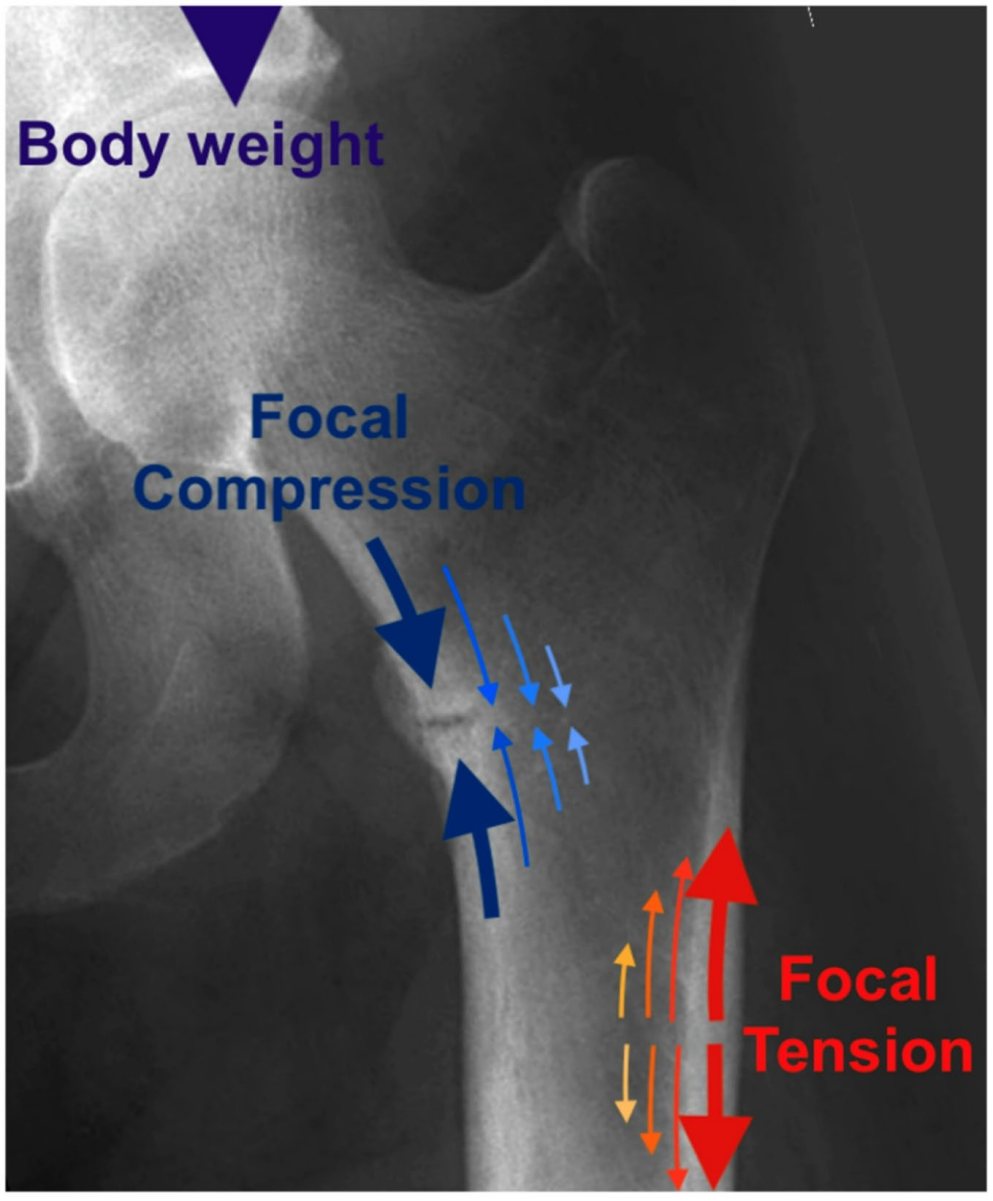
Forces in the femur during gait. **Dark blue arrows**. Classical **medial** femoral ‘Pseudofracture’ or ‘Looser zone’ in cortical bone in a 48-year-old male patient with severe vitamin D deficiency osteomalacia (but **not** HPP). The fracture line of osteomalacia typically occurs at a zone of high **compression** (during gait) in the medial femur. Such fractures are painful but rarely complete. **Dark red arrows.** Although unaffected in this patients’ femur, the red arrows indicate the typical location of highest **tension** (during gait) in a femur. This lateral cortical site is the stereotypical location of ‘brittle’ fractures which frequently give way to require surgical nailing in hypophosphatasia, genetic osteopetrosis and rarely in long term bisphosphonate users

**Fig. 2:**
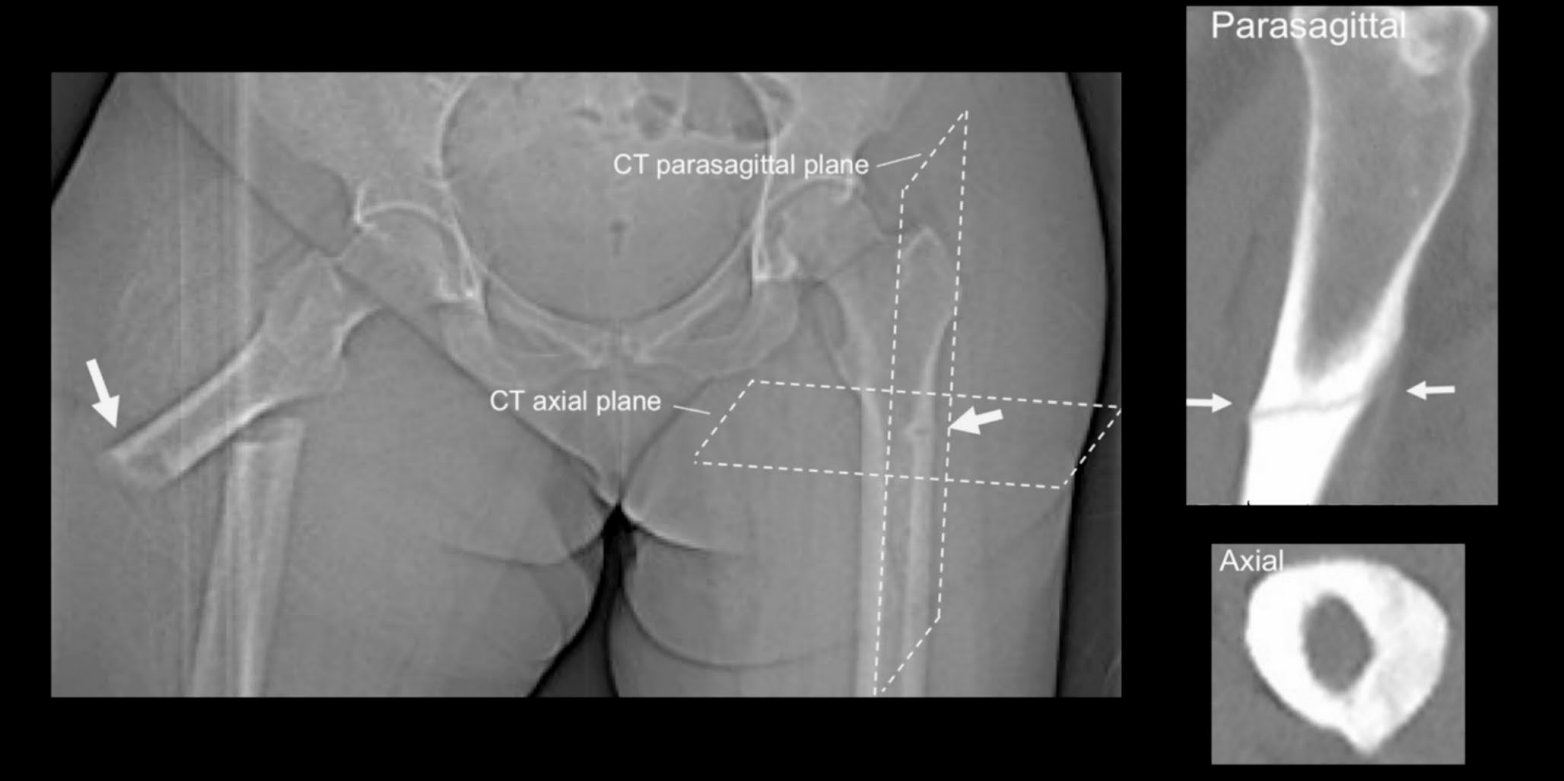
Classical lateral cortical bone fractures in HPP (a) CT scout view (left, main panel) of a 45-year-old female HPP patient whose first presentation was with a spontaneous ‘tensile’ fracture through her dense, thickened right femoral cortex. She was also found to have a left femoral endosteal thickening (arrowed). This endosteal expansion/callus seems widespread in this type of HPP fracture and is noted to be different to the periosteal ‘beaking’ of bisphosphonate-associated fractures. (b) Closer investigation of the left femoral cortex with oblique parasagittal (upper right) and axial (lower right) CT reconstructions showed an impending break with a large plateau of disconnected lateral femoral cortex. White arrows also show the extent of the macroscopically visible tensile fracture lines. Genetic analysis revealed compound heterozygosity at the ALPL gene (NM_000478:5: c.340G > A p.(Ala114Thr) and c.1363G > A p.(Gly455Ser))

This review will revisit a paradox in HPP research identified half a century ago; namely the differences in both the mechanism and anatomical site of femoral stress fractures in adult HPP compared to those observed in adult osteomalacia [7–9]. Bone pathology at the tissue level in HPP has been widely described as osteomalacia (i.e. soft bone), and yet the actual appearance of HPP femoral fracture is more in keeping with osteopetrosis (i.e. hard bone), which causes cracking, with sudden brittle failure. Fractures of the femur through soft, poorly mineralised bones in adults with osteomalacia tend to be incomplete, so-called pseudofractures, often at the medial femoral neck and medial shaft. Conversely, the mechanism of HPP femoral fractures is a slowly evolving lateral crack or split in the tensile femoral cortical shaft, which often presents as a spontaneous complete break. Such stress fractures are more in keeping with genetic osteopetrosis or the brittle tensile fractures sometimes observed in two unusual iatrogenic clinical scenarios: (i) in the dense, highly mineralised bones of long-term bisphosphonate users with impaired osteoclast function and (ii) in the femoral shaft of patients with non-endemic skeletal fluorosis [10]. Interestingly there is also excessive osteoid in biopsies of the latter [10]. We challenge the notion that the histological osteomalacia of HPP is solely responsible for the pathological femoral fractures seen [11]. Radiographs (Fig. 1) clearly illustrate the key differences [12] between the medial anatomical location of classical osteomalacia ‘pseudofractures’ and the lateral femoral stress fractures associated with HPP (Fig. 2). This distinction will be further explored in the ‘Clinical and radiological aspects’ section below.

## Genetics and Prevalence of Moderate Clinical Severity HPP with Relevance to Femoral Fractures

Although HPP was first described in 1948 by John C. Rathbun in a severely affected infant boy with rickets, seizures, and reduced serum TNAP (commonly known as alkaline phosphatase) activity [13] many studies have since been conducted to gain a deeper understanding of its complex genetics and underlying mechanisms. Wider use of genetic testing and improved clinical awareness have favourably impacted our appreciation of the epidemiology and clinical diversity of the disease. The condition exhibits a spectrum of severity and clinical forms have been variously classified based on the age of onset and the presence or absence of bone symptoms: perinatal, prenatal benign, infantile, childhood/juvenile, adult and odontohypophosphatasia [6, 14] Fortunately, the more severe forms remain rare [14, 15]. HPP genetic composition suggests a nosology with 3 clinical forms: severe HPP - recessive and rare; moderate clinical severity HPP - recessive or dominant, and a milder ‘carrier state’ - commoner and characterised by low TNAP and non-specific, if any, clinical signs [16]. Prevalence was estimated in Canada in 1957, where the clinically severe form was estimated to affect 1:100,000 live births [17]. More recently, the adult forms of HPP were found to have a prevalence of 1:508 to 1:3100 in Europe, while the more severe form affected 1:300,000 live births [16].

The biochemical hallmark of HPP, i.e., persistently low levels of TNAP reflects loss-of-function variants within the gene that encodes this enzyme, the *ALPL* gene [1]. Four genes encode alkaline phosphatase three of which are tissue-specific, i.e., intestinal, placental and germ cell (*ALPI*,* ALPP* and *ALPPL2*, respectively). The *ALPL* gene is in chromosome 1 and encodes the TNAP protein, present not only in bone, liver, kidney and developing teeth, but also in the central nervous system, fibroblasts, and other cells [6]. Currently, more than 480 variants have been described (https://alplmutationdatabase.jku.at/), 70% of which are missense [18]. Earlier-onset ‘severe’ HPP disease tends to have an autosomal recessive inheritance pattern [14]. Adult-onset disease may be inherited in either an autosomal recessive (including compound heterozygous) or an autosomal dominant (AD) fashion [2]. Presenting with spontaneous femoral fractures, confirms moderate clinical severity and where we have encountered the archetypal femoral fracture (including the patient shown in Fig. 2) we have tended to find heterozygous, biallelic variants. In terms of nomenclature, we do recognise that other authors have tended to refer to these patients as having a ‘mild’ form of disease [19], while others have referred to similar patients as having ‘severe’ HPP [20]. Added to the confusion is that *individual alleles* involved in HPP causation have been described as moderate, severe or mild [21].

Occasionally, adults present with autosomal dominant moderate clinical severity HPP through sustaining spontaneous lateral femoral fractures [8, 22]. Indeed, 7 missense variants affecting TNAP structure at the dimerisation site of the enzyme are now known to partly explain this dominant form of the disease [22], if not fully explaining variable expressivity and incomplete penetrance [8, 23]. Such incomplete penetrance and variable phenotypic expression are common in HPP, with different phenotypes occurring even among family members with proven compound heterozygosity and information on predicted enzyme function. Other ‘mild’, but symptomatic autosomal dominant forms can result from monoallelic inheritance of a pathologic single allele, and in some cases a poorly functioning TNAP leads to a haploinsufficiency state [19]. Where TNAP is less severely impaired by a single variant away from such important functional sites, a ’recessive carrier’ state can explain low TNAP levels due to a single *ALPL* variant [24]. Of relevance to this review, such ‘recessive carriers’ are certainly at risk of spontaneous femoral fracture in situations when a ‘second hit‘ (such as commencing bisphosphonates) uncovers a true HPP disease phenotype [20]. To the uninitiated, it might seem that there is a blurring of the lines between the various carrier and disease states, but diagnostic criteria are available [15]. This is important since in many cases, low serum alkaline phosphatase levels are found incidentally during routine blood tests or when an individual is tested following a diagnosis in a direct family member. Current diagnostic criteria for HPP as a disease remain primarily based on the combination of clinical manifestations, radiological findings, and laboratory abnormalities [15]. Persistently low serum TNAP levels (adjusted for age and sex) along with elevated levels of vitamin B6 and urinary phosphoethanolamine (PEA) corroborate the clinical diagnosis of HPP (Table 1). Genetic testing helps to confirm the diagnosis and is important for family counselling [25].

## Clinical and Radiological Aspects of Adult HPP Versus the Femoral Fracture of Osteomalacia

The clinical severity of HPP is usually, but not always [23], related to the degree of residual TNAP activity [6]. Moderate clinical severity adult HPP is typically identified in mature adulthood after sustaining unusual femoral or metatarsal fractures, although some patients are only identified after incorrect diagnosis of osteoporosis following various low trauma fractures [26]. Key HPP disease phenotypes of TNAP dysfunction (Table 1) are femoral, metatarsal and other unusual fractures, chondrocalcinosis, ectopic calcification of ligaments and pseudo-gout attacks due to calcium pyrophosphate dihydrate deposition [6, 27]. Hyperphosphataemia and hypercalciuria may also be observed as well as renal abnormalities such as nephrocalcinosis [4, 6]. A history of early loss of primary dentition (odontohypophosphatasia) is also common [6, 8].

**Fig. 3:**
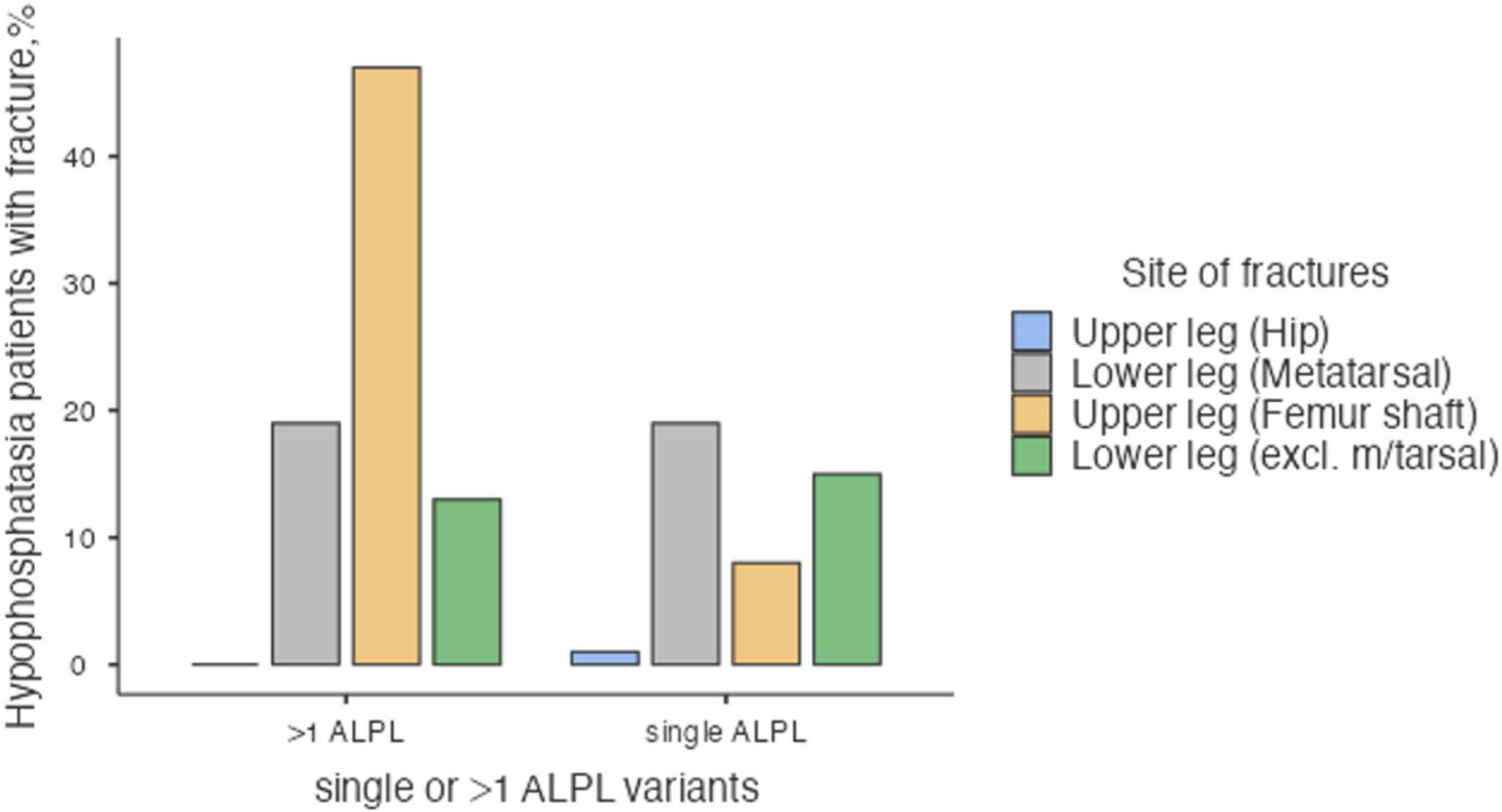
Adapted from Global hypophosphatasia registry data. Almost half (47%) of adult HPP patients with > 1 ALPL variant sustained subtrochanteric femoral shaft fractures compared with only 8% of those with a single ALPL variant. The femoral shaft was the only fracture type that showed genotype-phenotype interaction [28]

It has long been recognised that adults with HPP disease have both femoral and metatarsal fractures, but fewer vertebral fractures, compared to those with low bone mass [26]. Recent results from the Global Hypophosphatasia Registry confirmed these clinical findings by examining fracture patterns among 389 untreated adults, of whom 249 had sustained fracture [28]. Femoral shaft and metatarsal fractures were indeed commonest (Fig. 3), reproduced from Seefried et al. [28], and an intriguing genotype-phenotype interaction was identified, whereby having bi-allelic variants in *ALPL* predisposed the patients to an unusually high rate of subtrochanteric femoral fractures. In fact, almost half (47%) of adult HPP patients with > 1 *ALPL* variant sustained subtrochanteric femoral shaft fractures compared with only 8% of those with a single *ALPL* variant (Fig. 3). No other fracture showed this large imbalance. In younger adults (≥ 18 to < 50 years old) fractures in the lower extremities (e.g., femur, metatarsals) were also somewhat more common with more than 1 *ALPL* variant than in those with 1 *ALPL* variant. The study also confirmed the very low rate of vertebral fractures in older adults with the disease.

Femoral stress fractures in HPP have relatively few features in common with those seen in osteomalacic conditions. As shown in Fig. 1, adults with osteomalacia of various kinds typically present with incomplete, femoral stress pseudofracture lines in the medial compression cortex (of the femoral neck or shaft), usually after a prodrome of limping and bone pain [29]. Osteomalacia fracture lines arise in medial areas of soft bone subjected to repetitive compressive strain [12]. A ‘Looser’ radiolucent zone (named after the Swiss physician) describes the radiographic appearance of the transverse lucency (Figs. 1 and 2), perpendicular to the long axis of the bone [20]. Osteomalacic Looser zones are seen in the femur and metatarsal bones but can occur in other bones [6, 30]. In HPP femoral stress fractures present differently (Fig. 2); patients frequently sustain complete, tensile (Fig. 2) fracture at a lateral location of the subtrochanteric femoral shaft, and the cortical bone of the shaft often appears thickened [8]. This spontaneous and seemingly brittle mechanism of femoral fracture in HPP is not anticipated based on previous histological studies in HPP bone [31], a paradox which has long been recognised [7, 8]. In fact, there is poor understanding of the focal pathology and mechanisms that lead to spontaneous lateral femoral stress fractures in HPP. Bone samples from hypophosphatasia patients do demonstrate pathological osteomalacia, characterised by soft, poorly mineralised bone [31]. Yet, the actual presentation of femoral fractures in HPP is more akin to osteopetrosis or pyknodysostosis, which is associated with hard bone that is prone to cracking and sudden, lateral, brittle failure of the femur in tension [9]. A number of observers have highlighted similarities between HPP femoral fractures and the so-called ‘atypical’ lateral femoral fracture associated with bisphosphonate use [7, 32]. While medication related [20] occur in the same subtrochanteric location, they also have subtle differences on radiography [33]. Nevertheless, HPP stress fractures do align much more closely with those seen in the highly mineralised bones of long-term bisphosphonate users (associated with impaired osteoclast function) than those seen in osteomalacia [7, 8, 32]. The mechanism of femoral fractures in HPP appears to involve a slowly evolving lateral tensile crack or split in the femoral cortical shaft, frequently resulting in a spontaneous complete break [34]. While other monogenetic and osteomalacic bone disorders have occasionally been linked with unusual femoral fractures [35, 36], including the aforementioned lateral cortex fracture in osteopetrosis [9, 37], the gross morphological femoral changes in osteomalacic conditions do not really resemble the HPP-related lateral femoral fractures described here.

## Orthopaedic and Medical Treatment of the Femoral Fracture Site in Moderate Clinical Severity Adult HPP

Since the femoral fractures in HPP are often bilateral and begin as ‘stress fractures’, evolving until complete and sudden breaks occur, they require prophylactic intramedullary nailing or open surgical repair during acute orthopaedic admissions [25, 38]. Management of femoral fractures including the patient in Fig. 2 was via intramedullary nailing. Since the pathology often occurs in a ‘mirror image’ location in the contralateral proximal lateral femur, the procedure often needs to be bilateral, even if the stress fracture line in the contralateral femur has yet to fully break. Although our patient declined enzyme treatment, some patients with moderate clinical severity HPP are treated with asfotase alfa, demonstrating good symptomatic responses, as per a recent series that included one of our patients with compound heterozygous variants in ALPL and spontaneous bilateral femoral fractures [39]. We consider that the cost and unknown risks of long-term enzyme replacement therapy mean this treatment should be reserved for at least moderately affected adult patients where highly specific HPP disease features and signs match both biochemical abnormalities (such as low TNAP levels) and appropriately interpreted genetic results. Several case reports have demonstrated that teriparatide (parathyroid hormone 1–34) has been associated with improvements in bone turnover markers and increased TNAP activity, at least during the period of treatment [40]. Furthermore, it effectively enhances pain relief, mobility, and healing of atypical femoral fractures and pseudofractures in adults with HPP, serving as a viable therapeutic option for select patients [41] Recently, gene therapy has shown promising results to treat HPP [42].

## Bone Biopsy and Bone Density Studies in Moderate Clinical Severity Adult HPP

The bone tissue-level defect in HPP is usually described as osteomalacia (i.e., reduced bone mineralisation and accumulation of unmineralised osteoid, or hyperosteoidosis) with biopsy studies identifying osteomalacia features histologically [3, 31]. As we have outlined, osteomalacia alone does not seem to account for the femoral shaft fractures that occur in these patients. It is possible that pathologies differ by site. Osteomalacia changes in HPP account more for the incidence of generalised bone pain in the disease (and its response to TNAP enzyme replacement therapy) as well as the softening of bone in the metatarsals, tibia and ribs, the latter causing such difficult respiratory problems in infants with severe forms [2].

Studies have shown that adults with HPP can exhibit variable bone mineral density (BMD) and T-scores when assessed by dual-energy X-ray absorptiometry (DXA) or quantitative computed tomography (QCT) meaning that fracture risk estimation should not be based solely on DXA parameters [43, 44]. All of our HPP patients with moderate clinical severity disease e.g. those with femoral fractures due to bi-allelic variants in *ALPL*, have abnormally high spinal BMD by DXA [45]. The case in Fig. 2 spine had the lowest spinal BMD Z-score of these patients, at + 2.2 by DXA. Lumbar spine Z-score elevation in adults with HPP who experience fractures, is considered a marker of disease severity [20, 26, 46]. In contrast, femoral BMD is not generally elevated, nor a surrogate for HPP severity [46].

In generalised (non-genetic) osteomalacia spine BMD is reduced, increasing rapidly with treatment [47]. Inexplicably high vertebral BMD in HPP patients with lateral femur fractures therefore points to some perturbation of control over osteoblastic and/or osteoclastic processes in the axial skeleton. Intriguingly, not only HPP patients but also adults with genetic osteomalacia (XLH) can have greatly increased spinal BMD; corroborated as being truly elevated density by 3D QCT evaluation of trabecular bone [31, 48]. Our QCT studies indicate the same phenomenon in adults with moderate HPP [45]. Raised spine BMD scores were also noticed in children with X-linked hypophosphataemic osteomalacia (XLH) [44]. Such studies add credibility to the claim that raised vertebral trabecular bone volume is not just ‘artefact’ due to common para-vertebral, ligamentous or entheseal calcification in HPP affecting the measurement technique [48].

In the ilium, Barvencik et al. found increased osteoblast surface and well-maintained mineralised bone volume histologically in biopsies of 8 HPP patients when compared with equal numbers of vitamin D deficient or XLH patient biopsies [31]. Earlier, Fallon et al. had reported generally well-maintained iliac bone volume (BV/TV) despite histological osteomalacia in the age range 16–23 [3, 49]. Generalised (non-genetic) osteomalacia cases also show normal bone volume histologically [50], even when bone density by DXA or QCT is very low. Our data from several iliac crest biopsies of moderate clinical severity HPP patients (all with bi-allelic variants and lateral femur fractures) indicate even higher iliac mineralised bone volume that could be described as osteosclerosis [45, 51]. It seems that some unknown mechanism seems to increase axial trabecular volume and results in higher spinal/iliac BMD even in the presence of tissue level hyperosteoidosis and osteomalacia [48] This might involve elements of osteoblast activation [31] and/or osteoclast absence or dysfunction [3] in adult HPP which may be of relevance to the AFF-like femoral fractures in HPP.

**Fig. 4:**
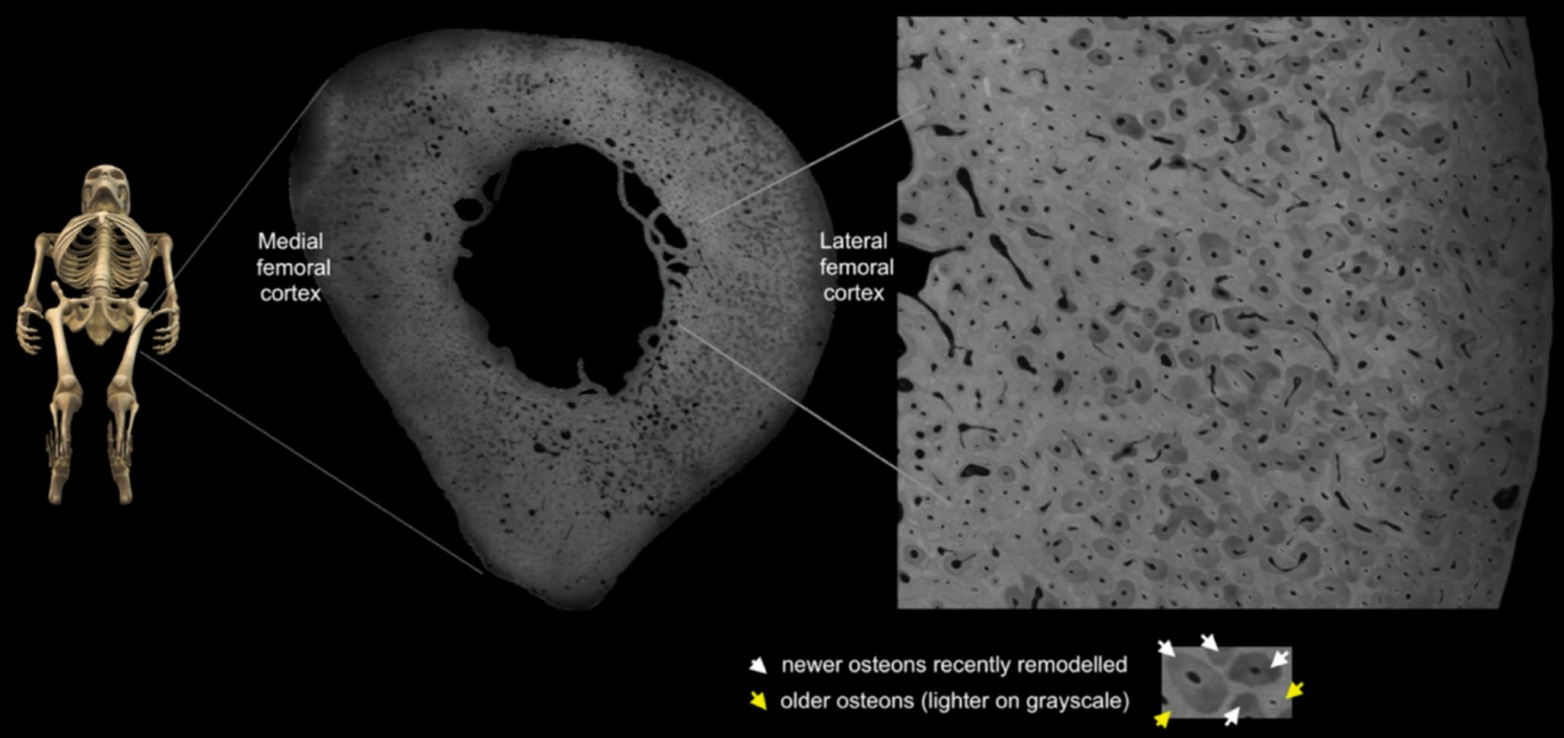
The normal lateral subtrochanteric femoral cortex is a site of extensive new osteonal remodelling/ resorption throughout life in response to tensile microdamage. A polished, contact femoral microradiograph of a male aged < 25 years with permission from C.D.L.T, courtesy of the Melbourne Femur collection. The extensive newer osteons are darker grey compared to the established, lighter grey older osteons [59]

**Fig. 5:**
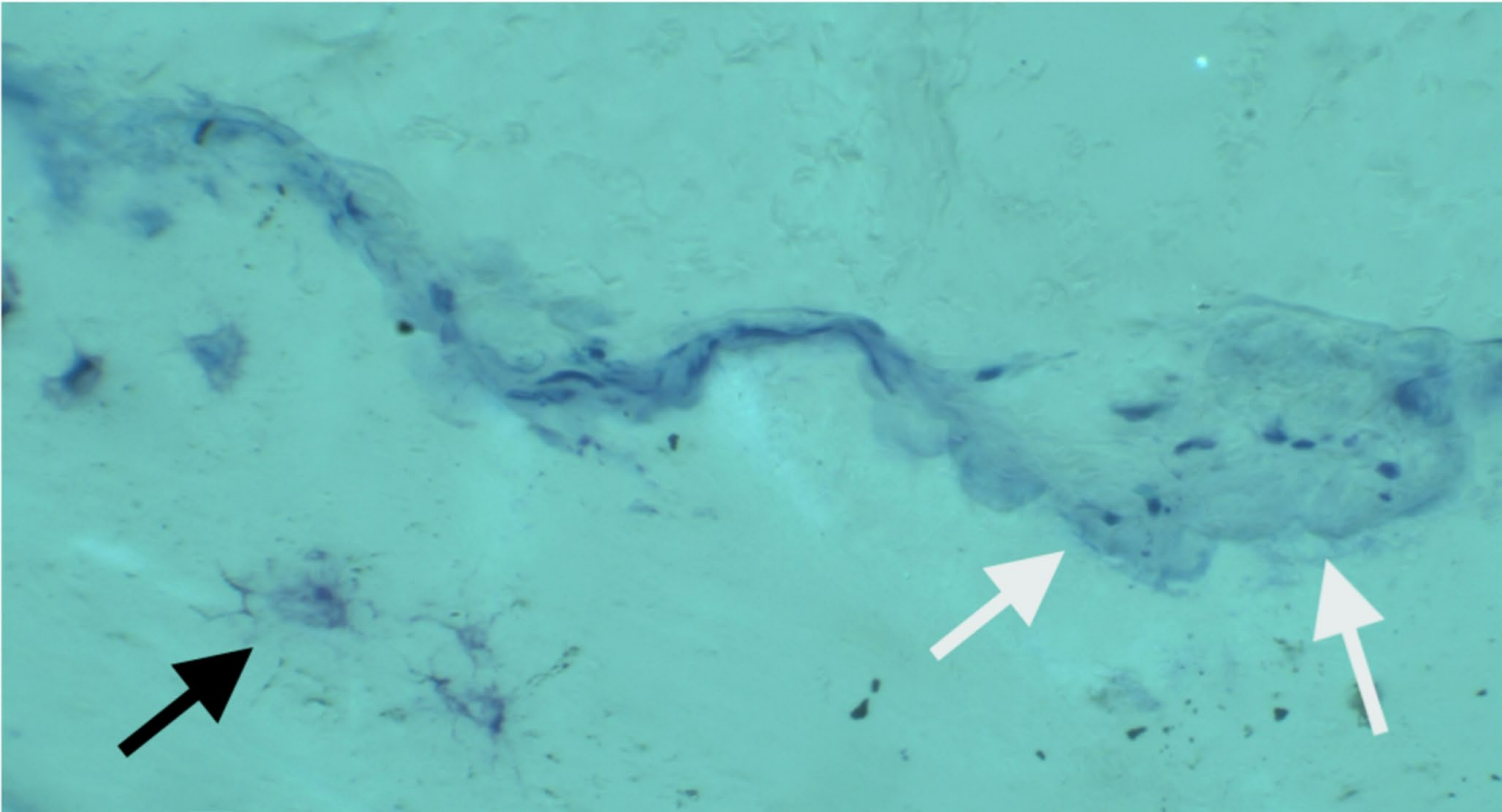
Toluidine blue stained undecalcified section of the periosteal surface of an iliac bone biopsy from a patient with hypophosphatasia. She has compound heterozygous mutations in ALPL and was admitted with bilateral, spontaneous femur fractures, like the patient in Fig. 1. Multinucleated osteoclasts are present, adjacent to recently resorbed mineralised bone (white arrows). Howship’s lacunae are excessively ‘scalloped’ or ‘jagged’ at the edges which has been noted previously in HPP bone. There is normal bone cell morphology, including osteocytes in the lacunocanalicular network (black arrow). A 6-layer composite Z-stacked image using 40x lens magnification with crossed polarising filters to highlight bone tissue

The prevailing, repetitive mechanical tensile forces in the normal subtrochanteric femoral shaft during walking direct osteoclastic cutting cones to extensively and repeatedly remodel microdamage throughout life (as indicated by the density of overlapping Haversian canals in the microradiograph from a young male in Fig. 4). It is also known that the disease analogues of HPP lateral femoral fractures, namely genetic osteopetrosis and bisphosphonate-associated AFF, are caused by (i) osteoclast abnormality, absence or dysfunction, and (ii) abnormally increased bone mineralisation or by failure of osteoclasts to resorb focally micro damaged bone [52]. So, in the HPP lateral femoral cortex, we envisage a local pathological process encompassing reduced osteoclastic resorption, abnormal mineralisation, osteosclerotic effects and abnormal healing responses to microdamage. Bone tissue is intrinsically weaker in tension than compression [53]. Normally, micro- and macroscale cracks in the lateral femur are resorbed and repaired through the actions of longitudinal osteoclastic tunnelling and/or callus formation responding to osteocytic signals (Fig. 4). In bisphosphonate-related atypical lateral tensile fractures of the femur, careful histological evaluation uncovered relevant pathologies including woven bone and amorphous material between the opposing edges of the crack, and the hypothesis was put forward that repetitive tensile stress prevented cellular repair mechanisms [52]. Although such studies are difficult and the chances for sampling are few, we and others have begun to explore the phenomenon by both bone biopsy methods (Fig. 5) and high-resolution analysis of the material properties of HPP bone specimens [45].

An important clinical practice point is that while low BMD is not a characteristic feature of HPP [54], adult HPP carriers risk being diagnosed with DXA-defined osteoporosis if they undergo femoral neck DXA after breaking a bone. In a metabolic bone clinic population seeing both HPP and fragility fracture patients, the HPP population were younger, and predictably had more metatarsal and femoral shaft fractures plus fewer vertebral fractures [26]. The inadvisable administration of bisphosphonates to HPP carriers, on the premise that such patients have osteoporosis, is one well-recognised precipitant of lateral tensile femur fractures. This phenomenon also implicates the osteoclast dysfunction axis and/or increased bone tissue mineralisation in the genesis of femoral fractures in HPP [55]. Interestingly, as the problem of bisphosphonate-associated AFF in osteoporotic patients grew in the literature, so did case series demonstrating such fractures among patients with HPP, few among whom had been treated with bisphosphonates [36, 38, 56–58]. In the next section, we explore what is known about the biomineralisation of bone in the disease, considering these observations.

**Table 1:** Main clinical and biochemical manifestations of HPP. ALP: alkaline phosphatase, PLP: pyridoxal 5’-phosphate, PEA: phosphoethanolamine

Clinical	Biochemical
*Skeletal*	*Age and sex adjusted ALP (serum)*
- Fractures (mainly metatarsal and femoral)	- Low
- Pseudofractures	
- Rickets	*Calcium (serum)*
- Bone deformities	- Elevated in severe cases
- Craniosynostosis	
*Dental*	*Phosphate (serum)*
- Premature loss of decidual and permanent teeth	- Elevated in severe cases
*Articular*	
- Chondrocalcinosis	*PLP / vitamin B6 (serum)*
- Pseudogout/crystal arthropathy	- Elevated
- Osteochondral spurs	
*Muscular*	*PEA (urine)*
- Chronic muscle pain	- Elevated
- Reduced muscular strength and performance	
- Enthesopathy	
*Renal*	
- Hyperphosphatemia	
- Hypercalciuria	
- Nephrocalcinosis	
*Neurological*	
- Seizures	
- Craniosynostosis-related symptoms	
*Respiratory*	
-Respiratory complications and failure (related to chest deformities)	

## Biomineralization of Hypophosphatasia Bone

Biomineralization of the skeleton is a tightly regulated process in which bone tissue is produced by osteoblasts. It is dependent on the complex interplay between calcium (Ca^2+^), inorganic phosphate (Pi) and pyrophosphate (PPi), hormones such as parathyroid hormone (PTH), vitamin D, fibroblast growth factor 23 (FGF23), collagen [60, 61] and enzymes including TNAP [6].

**Fig. 6:**
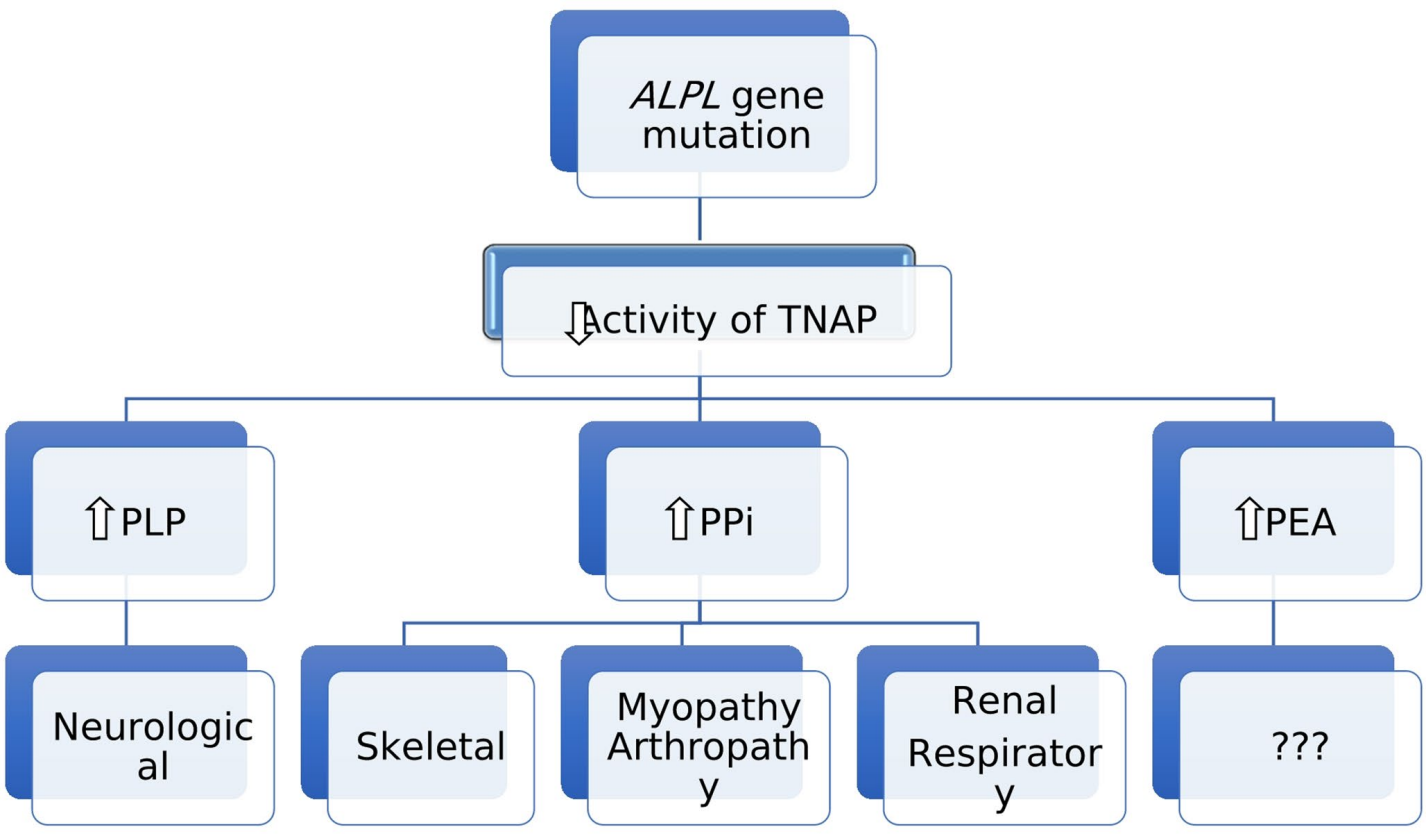
Multisystem involvement in HPP. TNAP: tissue-nonspecific alkaline phosphatase, PLP: pyridoxal 5’-phosphate, PPi: inorganic pyrophosphate, PEA: phosphoethanolamine. Adapted from Conti et al., 2017 [4]

TNAP is a glycosylphosphatidylinositol (GPI)-linked cell-surface enzyme which exhibits phosphatase activity towards several substrates. In HPP, the reduced activity of TNAP may lead to accumulation of three main metabolites, *i.e*, pyridoxal 5’-phosphate (PLP) which is the major circulating form of vitamin B6; inorganic pyrophosphate (PPi); and phosphoethanolamine (PEA) [25] (Fig. 6). More recently, it has been suggested that adenosine triphosphate (ATP) [62], di-phosphoryl lipopolysaccharide (LPS) [63] and phosphorylated osteopontin (p-OPN) [64] are also substrates of TNAP. Impairment of PLP dephosphorylation may cause pyridoxine-dependent seizures, while PPi accumulation is widely postulated to inhibit bone mineralisation. The paradoxically raised bone density in the patients remains unexplained. The consequences of PEA accumulation remain unknown although recent studies have demonstrated its utility as a diagnostic biomarker for HPP as well as for monitoring treatment response [17, 65].

The key roles of TNAP in bone and tooth mineralisation have been elucidated experimentally. In the skeleton, TNAP is located on the cell surface of osteoblasts and chondrocytes, including their shed matrix vesicles (MVs) [66]. MVs are nanoscopic extracellular vesicles which are implanted within osteoid by mineralising cells and are hypothesised to drive accumulation of calcium phosphate in amorphous non-crystalline states through sophisticated biochemistry mediated in part by TNAP [67–69]. In the normal process of biomineralization, TNAP hydrolyzes pyrophosphate, PPi, in the extracellular space into inorganic phosphate which is transported intravesicularly by phosphate transporter-1 (Pit-1) [67, 68].Simultaneously, Pi is generated by another enzyme inside MVs, the orphan phosphatase 1 (PHOSPHO1), through hydrolysis of phosphocholine and PEA, derived from phospholipid headgroups in the vesicle membrane [70]. Transport of amorphous calcium phosphates from the internal vesicular environment to collagen fibrils and the chemistry governing their penetration of and nucleation within fibrils remains the subject of much debate in the literature, but is likely governed in part by acidic non-collagenous polyphosphorylated proteins, including p-OPN [71]and poly (ADP ribose), a highly phosphorylated protein post-translational modification upregulated in bone mineralization [72, 73]. The critical function of TNAP in biomineralization of the skeleton is demonstrated by the mouse model in which genetic ablation of *ALPL* induced a phenotype mimicking infantile HPP, with severe skeletal abnormalities. The hypomineralized phenotype was attributed to the lack of TNAP-mediated PPi hydrolysis, consequential accumulation of PPi and the inability of the mineral phase to propagate in the presence of excess PPi. Simultaneous abolition of PHOSPHO1 in the same model resulted in a complete absence of mineralization in the embryonic skeleton and dentition [70].

The normal physiological bone mineralization process results in a poorly-crystalline carbonated calcium phosphate mineral, similar to the inorganic mineral hydroxyapatite, embedded within and around collagen I fibrils. The mineral takes the form of thin apatitic platelets (1–2 nm in width), surrounded by hydrated, amorphous hydrogen phosphate-rich mineral, the latter likely also containing large metabolite anions including citrate and lactate which help to maintain the amorphous regions between neighbouring apatitic platelets, preventing these delicate structures from collapsing. The physical chemistry governing the transformation of amorphous phases generated by MVs to create this complex molecular architecture has yet to be elucidated, however the local availability of both Pi and of mineralization perturbers such as PPi is likely to be crucial in calibrating these physical dynamics. The presence of TNAP creates Pi through hydrolysis of PPi to two Pi, whilst lack of TNAP allows PPi to persist. TNAP is therefore hypothesised to establish a Pi: PPi ratio permissible for driving the physical chemistry equilibria towards this intricate nanostructure. PPi is generated in the extracellular space by the membrane-bound enzyme ectonucleotide pyrophosphatase/phosphodiesterase 1 (ENPP-1) through the hydrolysis of ATP [40]. So both TNAP and ENPP-1 are regulators of extracellular PPi [74, 75]. The perturbing effect of PPi on biomineralization is thought to be achieved by its binding to nascent crystal surfaces, slowing mineral crystal growth from those crystal surfaces. PPi has a well-established function in inhibiting calcification in soft tissues, leading to PPi being categorized a “mineralization inhibitor”. The discovery of PPi in the bone mineralization system inspired the synthesis of the bisphosphonate drug family, which similarly bind hydroxyapatite and are in widespread use for the treatment of low bone mass conditions such as osteoporosis due to their inhibitory effect on bone resorption [76, 77]. However, in the highly complex chemical milieu of mineralizing bone, PPi may be better understood as a perturber of the normal physical chemistry pathway that leads to the proper nanoarchitecture of bone mineral, rather than as an agent that prevents mineral formation.

Another molecule widely described as a potent mineralization inhibitor, p-OPN, binds to hydroxyapatite as soon as it is exposed to the extracellular fluid, thus contributing to the regulation of the degree of extracellular matrix mineralization [7]. Absent TNAP function leads to accumulation of p-OPN, suggesting that OPN is another substrate for TNAP. Indeed, both elevated levels of PPi and phosphorylated OPN are found in mouse models of HPP [64, 75]. Overall, a decrease of TNAP reduces Pi availability for bone mineralization, while increasing the levels of PPi and p-OPN, which likely inhibit normal mineralization routes. Serum phosphate tends to be elevated in HPP [78].

HPP is an unusual type of osteomalacia given that circulating levels of calcium, phosphate and vitamin D are not low, but instead, there is an inhibition of the mineralization process itself [6]. Given that osteomalacia from PPi accumulation does not fully explain the pathophysiology of lateral femur fractures in the disease, research should be directed at understanding the complex molecular structure of the diseased calcified tissue and understanding the physical chemistry of how it is formed. HPP patients have highly mineralized bones, as evidenced by their high BMD, so accumulation of PPi through lack of TNAP function is clearly not preventing mineral formation. More likely, accumulation of PPi perturbs the delicate chemical equilibria involved in forming the complex nanoscopic bone mineral architecture so research on any differences in bone mineral architecture in HPP patients to that described above would be valuable.

Exploring the relative contributions of abnormal cellular signalling and activity alongside impaired bone mineralization in HPP seems a worthwhile strategy, to help understand and prevent macroscale crack initiation and propagation in patients’ femora. Preliminary microscopic studies indicate plentiful osteoclasts in compound heterozygous HPP alongside so-called ‘scalloping’ phenomenon of the resorbed surface (Fig. 5). This suggests osteoclasts are present and functioning (unlike in tensile femur fractures from osteopetrosis or bisphosphonate overuse). Therefore, the intrinsically brittle bone in HPP looks more likely as a cause of tensile fatigue damage, so a detailed analysis of the material properties and nanoscale crystal structure of the mineralised tissue in these patients is required.

## Data Availability

No datasets were generated or analysed during the current study.
